# Evaluating Public Sentiment on Attention-Deficit/Hyperactivity Disorder and Autism Spectrum Disorder Compared With Other Mental Health Disorders From Posts on X (Formerly Known as Twitter): Longitudinal Analysis

**DOI:** 10.2196/74440

**Published:** 2026-04-09

**Authors:** Vitor Tiosso Batistetti, Facundo G Sanchez, Andrea Varaona, Francisco Lara-Abelenda, Mariana Pinto da Costa, Juan Pablo Chart-Pascual, Alberto Rodriguez-Quiroga, Javier Quintero, Miguel Angel Alvarez-Mon

**Affiliations:** 1 Universidad Abierta Interamericana Buenos Aires Argentina; 2 Department of Psychiatry Lincoln Medical Center New York, NY United States; 3 Department of Medicine and Medical Specialties Faculty of Medicine and Health Sciences University of Alcala Alcalá de Henares Spain; 4 Department of Signal Theory and Communications Rey Juan Carlos University Fuenlabrada Spain; 5 South London and Maudsley NHS Foundation Trust London United Kingdom; 6 Department of Child and Adolescent Psychiatry Institute of Psychiatry, Psychology & Neuroscience King's College London London United Kingdom; 7 BioAraba Research Institute Vitoria Spain; 8 Department of Psychiatry Hospital Universitario de Álava Vitoria Spain; 9 University of the Basque Country Vitoria Spain; 10 Department of Psychiatry and Mental Health Hospital Universitario Infanta Leonor Madrid Spain; 11 Department of Legal and Psychiatry Universidad Complutense de Madrid Madrid Spain; 12 CIBERSAM-ISCIII Biomedical Research Networking Centre in Mental Health Instituto de Salud Carlos III Madrid Spain; 13 Instituto Ramón y Cajal de Investigación Sanitaria Madrid Spain

**Keywords:** neurodevelopmental disorders, social media, public opinion, mental health, trivialization, Twitter, X, cross-cultural comparison, English and Spanish analysis

## Abstract

**Background:**

Neurodevelopmental disorders, especially attention-deficit/hyperactivity disorder (ADHD) and autism spectrum disorder (ASD), have seen a marked rise in public attention, yet research on public opinion remains limited. Social media analysis offers real-time, unfiltered insights into public perceptions, enabling empirical examination of public attitudes and opinions.

**Objective:**

This study aimed to assess the evolution of public opinion on ADHD and ASD between 2009 and 2023 by analyzing posts from X (formerly known as Twitter; X Corp), comparing perceptions across English and Spanish languages and against other mental health conditions.

**Methods:**

Posts mentioning keywords related to ADHD and ASD and control conditions (eg, depression, anxiety, insomnia, bipolar disorder, schizophrenia, suicide, and substance use disorders) were collected from X between 2009 and 2023. The dataset included posts in both English and Spanish. Machine learning algorithms were then applied to classify post content into predefined categories, including volume of posts, engagement, personal experiences, trivialization, perceived causes, and perceived treatability. Parametric and nonparametric tests were used to assess for differences by language. Descriptive statistics were presented using tables and graphical representations.

**Results:**

A total of 852,990 posts were analyzed, including 511,510 (59.97%) in English and 341,480 (40.03%) in Spanish. Overall, post volume on mental health conditions increased across the study period. In English, posts about ADHD (97,084/511,510, 18.98%) and ASD (74,619/511,510, 14.59%) were among the most frequent, while of the 341,480 Spanish posts, there were 49,475 (14.49%) ASD posts, significantly outnumbering ADHD posts (n=18,223, 5.34%; chi-square test *P*<.001). Engagement analysis indicated a notable increase in likes and reposts per post over time, particularly after 2019, with ADHD-related posts in English experiencing peak engagement during the COVID-19 pandemic. However, ASD posts had comparatively lower engagement across languages. Posts sharing personal experiences were more polarized in Spanish, with higher proportions of negative and positive experiences compared with English posts. Trivialization of mental illnesses was less common in Spanish posts than in English posts, particularly for ADHD (17,053/18,223, 93.59%; chi-square test *P*<.001) and ASD (41,933/49,475, 84.73%; chi-square test *P*<.001). User-perceived causes included multifactorial factors, biological or genetic factors, substance use, psychological susceptibility, acute psychosocial stressors, and COVID-19. Perceived treatability varied by language but consistently included high perceived incurability, limited improvement despite professional help, and low perceived self-manageability except for anxiety.

**Conclusions:**

Analysis of social media discourse showed that ADHD attracted higher post volumes, particularly during the COVID-19 pandemic, often described with multifactorial causes including substance use and genetics. ASD consistently received lower engagement. Both language groups showed low trivialization, awareness of the chronicity of the illness, and limited support for the self-management of mental health conditions. These findings underscore social media’s value for capturing direct public perceptions to guide future educational and intervention efforts.

## Introduction

Neurodevelopmental disorders, such as attention-deficit/hyperactivity disorder (ADHD) and autism spectrum disorder (ASD), are complex conditions affecting communication, attention, and behavior [1]. Public interest in these disorders has surged in recent years, leading to more frequent diagnoses and widespread discussion about their nature [2]. Despite their increased visibility, public understanding remains limited and misconceptions persist [3-7]. Traditional survey methods, including structured questionnaires and qualitative interviews, while valuable, often fail to capture these nuanced perceptions [8,9]. ADHD is common with pooled global estimates of approximately 5% in children and recent US estimates of approximately 1 in 10 among school-aged children. ASD is also common, with global estimates of approximately 1% and recent US surveillance estimates of approximately 1 in 36 among children [10,11]. The existing literature suggests that the rise in ADHD and ASD diagnoses may be attributed to changes in diagnostic criteria and greater clinical recognition [2]. However, this may not translate into a broader public understanding of these conditions [3,4,7,12]. Traditional research methods, such as surveys, interviews, and focus groups, may not capture the full spectrum of public beliefs and discourse [8,9]. These limitations of survey-based approaches informed our research questions about how ADHD and ASD are discussed in more naturalistic, publicly expressed discourse on social media and whether patterns differ by condition and language. X (formerly known as Twitter; X Corp) provides a large, more diverse, and spontaneous sample in real time, offering insights without the constraints of structured methodologies [13]. This platform facilitates anonymous communication by eliminating the complexities of face-to-face interactions, including nonverbal cues [14]. By using advanced machine learning methodologies to analyze posts, it is possible to quantify and characterize trends in public discourse on the platform, providing an alternative approach to traditional survey-based assessments of public perception [6].

In this study, we addressed the existing gap in understanding platform-level public discourse on neurodevelopmental disorders by analyzing a large sample of posts collected over more than a decade. We compared English- and Spanish-language posts to examine whether discourse patterns differ across large language communities. We hypothesized that, despite higher post volumes and engagement, public discourse on ADHD and ASD remains mixed, varying by condition and language. Our analysis sought to elucidate current opinions and attitudes, thereby informing future strategies for education and interventions.

## Methods

### Data Collection

Posts were gathered using a search engine called Tweet Binder (SocialBro SL) [15] that has access to the totality of public posts, covering the period from 2009 to 2023. Posts were analyzed over this same period, reflecting the full time range available in the exported dataset for our query at the time of retrieval. The start year corresponds to the earliest period with consistent availability of posts and engagement metadata in the dataset [16]. The data collection focused on posts that mentioned the following keywords related to mental illness in both English and Spanish: *ASD/autism*, *ADHD*, *depression*, *bipolar* *disorder*, *anxiety*, *substance* *use*, *schizophrenia*, *insomnia*, *suicide*. The dataset included posts in English and Spanish from all locations. Metadata associated with each post, including the date, time, user location (if available), and user engagement metrics such as likes and reposts, were also collected. Engagement metrics included likes and reposts as provided by Tweet Binder; reply or comment counts were not available in the exported dataset and were not analyzed. The collected posts underwent filtering to remove duplicates, non-English or non-Spanish posts, and posts lacking substantial content (eg, posts with only hashtags, emojis, or single words). For clarity, we use the term “post” to refer to a unit of content on X (formerly called a “tweet” on Twitter).

### Ethical Considerations

This study was approved by the Research Ethics Committee of the Universidad de Alcalá (approval code: OE 14_2020). The study was conducted in compliance with the ethical principles of the World Medical Association Declaration of Helsinki (7th revision, 2013). As the study exclusively analyzed publicly available, anonymized posts from X with no direct interaction with or identification of individual users, the ethics committee determined that individual informed consent was not required and granted a waiver of informed consent. No compensation was provided, as no human participants were directly enrolled in this study. All data were anonymized.

### Machine Learning

Machine learning is essential for analyzing large datasets that are impractical to evaluate manually. As a subset of artificial intelligence, machine learning includes 3 main approaches: supervised, unsupervised, and semisupervised learning [17]. In this research, we used semisupervised learning, which combines elements of both supervised and unsupervised methods using both labeled and unlabeled data. This method extends traditional manual analysis, aiming to develop a model that replicates expert evaluations for classifying millions of posts.

The machine learning application begins with a preprocessing step where the posts are normalized by splitting negative contractions, removing special characters and repetitions, and converting emojis into text. Next, 2 manually classified datasets (1 in English and 1 in Spanish), each consisting of 1500 posts, were labeled by 2 study team annotators using a prespecified codebook; Spanish-language labels were completed by native Spanish annotators, and disagreements were resolved by consensus. We used 75% of each labeled dataset for training and 25% for testing, which were randomly divided into a 75% training subset (1125 posts) and a 25% testing subset (375 posts). The training subset was used to train a machine learning model for each classification category: *classification* (distinguishing posts relevant to mental health discussions from nonrelevant content); *trivialization* (identifying posts containing terms or expressions that minimize, dismiss, or undermine the seriousness, impact, or legitimacy of mental health conditions); *personal* *experience* (capturing firsthand accounts or self-reported experiences related to a mental health condition); *user-perceived treatability* (assessing whether a condition is viewed as incurable, treatable with professional help, or self-manageable, reflecting whether a post suggested that symptoms can improve with treatment including medication and psychosocial interventions); and *causes* (detecting attributions regarding the origins of mental health conditions, whether multifactorial, biological or genetic, substance related, or psychosocial). The testing subset was then used to validate the models’ performance.

Despite the availability of various pretrained models for text classification, we opted for the Transformer-Based Language Model Pretrained on English Posts (BERTweet) model [18] (VinAI Research) for the English dataset and Transformer-Based Language Model Pretrained on Spanish Text (BETO; Universidad de Chile) [19] for the Spanish dataset. BERTweet, trained on 80 GB of text containing more than 860 million English posts, was selected due to its extensive use in the literature [20,21] and its specific training on English posts similar to those we evaluated. For the Spanish dataset, we chose BETO, a Bidirectional Encoder Representations from Transformers model trained on a Spanish corpus, which is also popularly used in the literature [22,23].

To ensure these models accurately replicate expert analyses, fine-tuning was conducted for each category. This process involves adjusting the parameters of the pretrained models using data specific to the new task, leveraging the general knowledge acquired during pretraining on large, unlabeled datasets to adapt them for more specialized tasks. The Spanish dataset was used to fine-tune BETO, while the English dataset was used to fine-tune BERTweet. A common challenge during fine-tuning is the imbalance of options within each category. To address this, we used the Easy Data Augmentation (EDA) [24] pipeline to generate additional posts, ensuring balanced representation across categories. EDA generates new posts by replacing words with synonyms, randomly removing some words, and rearranging word positions. EDA was used only to augment the training data during model fine-tuning; synthetic EDA-generated posts were not included in the final analytic dataset or in results.

The performance of the fine-tuned models was evaluated using the test datasets, with the *F*_1_-score used to measure accuracy across all categories. The English dataset models achieved the following *F*_1_-scores: classification (0.78), trivialization (0.70), personal experience (0.69), user-perceived treatability (0.78), and causes (0.65). Similarly, the Spanish dataset models presented an equivalent performance: classification (0.79), trivialization (0.78), personal experience (0.69), user-perceived treatability (0.70), and causes (0.66). After confirming the models’ effectiveness, they were deployed to classify the remaining posts. First, the classification category was applied to both datasets, and only the posts classified as relevant were further analyzed across the other categories, whereas those classified as irrelevant were discarded. There is no universally accepted *F*_1_-score cutoff across tasks and class distributions; we report *F*_1_-scores to allow comparison across our models and interpret them in the context of class imbalance and the complexity of each label. We used supervised machine learning to classify posts into predefined content categories. Within the personal experience category, posts were additionally categorized by valence (negative, neutral, or positive). Negative personal experience posts included experiences such as stigma or discrimination or distress related to the condition.

### Descriptive Analysis

Descriptive statistics were presented both in tables and in different forms of graphical representation, including line graphs for the temporal evolution of post frequencies and clustered bar charts for comparing different categories of posts by language. These analyses were performed using the Python programming language (version 3.10.12; Python Software Foundation).

To assess the impact of ADHD and ASD on X, we analyzed the number of posts from 2009 to 2023 compared to other common psychiatric diagnoses. Specifically, we included anxiety disorders, schizophrenia, bipolar disorder, depressive disorders, substance use disorder (SUD), insomnia, and suicide.

Posts describing personal experiences were treated as one component of the broader public discourse and analyzed separately from general discussion themes. In this study, *public sentiment* refers to what people publicly say about these conditions on the platform, including the themes and attitudes expressed in posts and how widely posts are liked or shared.

### Statistical Analysis

Statistical analysis of the data was performed using both parametric and nonparametric tests to assess between-group differences in categories associated with mental illness on X by language. Initially, we verified the completeness of the data by confirming the absence of missing data on relevant variables.

To test for statistical assumptions, the Shapiro-Wilk test was used to assess the normality of the residuals, and the Levene test evaluated the homogeneity of variances between groups. As the assumptions required for ANOVA were not fully met, alternative methods were chosen. A simplified ANOVA was performed to confirm the initial findings, and the Kruskal-Wallis test was applied as a nonparametric alternative. The results were subsequently adjusted using the Benjamini-Hochberg method to control for the false discovery rate and the Holm method to adjust for the type 1 error rate. To further explore differences between specific groups within each category, the Dunn test was used for pairwise comparison.

Additionally, the chi-square test was used to determine whether the observed differences between the frequencies of categories associated with different illnesses by language and the expected frequencies were statistically significant.

## Results

### Number of Posts Related to ADHD, ASD, and Other Mental Illnesses

**Figure 1:**
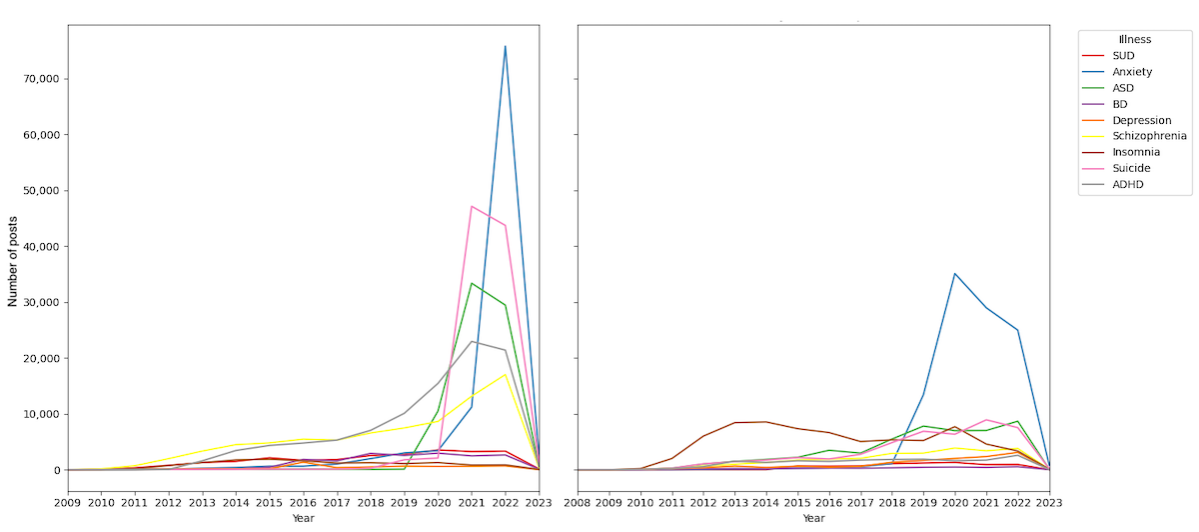
Annual number of posts by mental health condition. Left: posts in English. Right: posts in Spanish. ADHD: attention-deficit/hyperactivity disorder; ASD: autism spectrum disorder; BD: bipolar disorder; SUD: substance use disorder.

From the total dataset of posts across all conditions, 852,990 were deemed classifiable for analysis. Among these classifiable posts, 511,510 of 852,990 (59.97%) posts were in English, and 341,480 (40.03%) were in Spanish. Overall, the number of posts about mental health conditions increased over the years (Figure 1). In English, 97,084 of 511,510 (18.98%) posts were related to ADHD, and 74,619 (14.59%) posts were related to ASD, with ADHD posts significantly more frequent than ASD posts (chi-square test *P*<.001). In Spanish, ADHD-related posts totaled 18,223 of 341,480 (5.34%) posts, while ASD-related posts totaled 49,475 (14.49%), with ASD posts significantly outnumbering ADHD posts (chi-square test *P*<.001).

In English-language posts, the most mentioned conditions were anxiety (100,723/511,510, 19.69% of posts); ADHD (97,084/511,510, 18.98% of posts); and suicide (96,817/511,510, 18.93% of posts). In Spanish-language posts, the most mentioned conditions were anxiety (105,780/341,480, 30.98% of posts); insomnia (70,561/341,480, 20.66% of posts); and ASD (49,475/341,480, 14.49% of posts).

### Engagement Analysis

**Figure 2:**
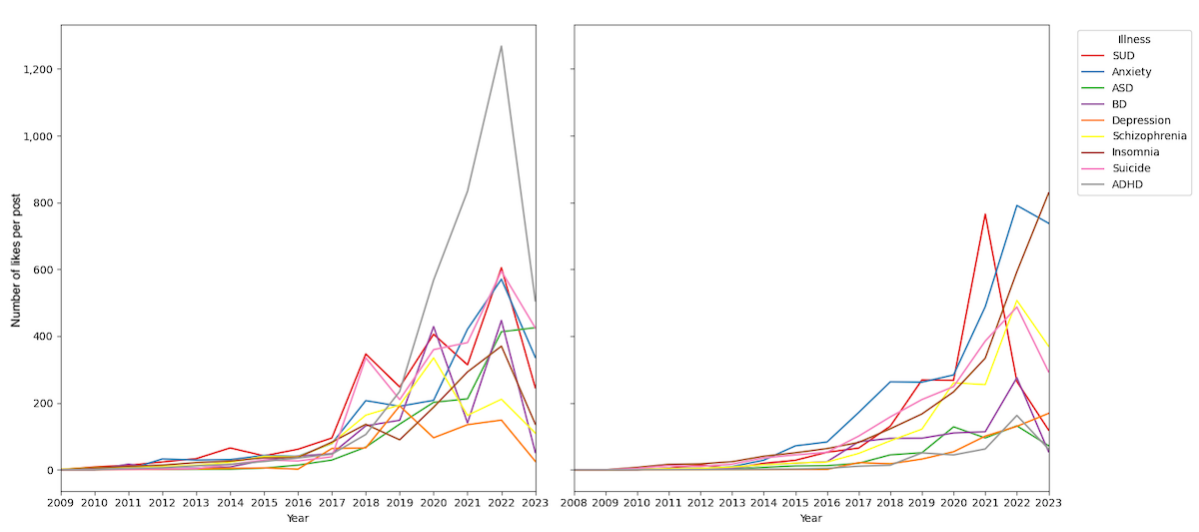
Line graphs illustrating the number of likes per post over time for various mental health conditions in both English (left) and Spanish (right). ADHD: attention-deficit/hyperactivity disorder; ASD: autism spectrum disorder; BD: bipolar disorder; SUD: substance use disorder.

Figure 2 presents line graphs illustrating the number of likes per post over time for various mental health conditions in both English- and Spanish-language posts. Overall, an increasing trend in likes per post was observed across conditions, particularly after 2019. Likes per post differed significantly across conditions in both English- and Spanish-language posts (*P*<.001 [Kruskal-Wallis test]). In English-language posts, ADHD-related posts experienced a significant increase in engagement, peaking around 2021, with likes per post reaching more than 1200. This period coincided with the COVID-19 pandemic. However, ASD did not receive the same level of attention as ADHD, with engagement levels remaining relatively low compared with other conditions. Across the full study period, ADHD posts had higher likes per post than ASD posts in both English and Spanish (both *P*<.001 [Kruskal-Wallis test]). Among the other conditions, anxiety, SUD, and suicide maintained steady engagement over the years, with several conditions surpassing 500 likes per post at their peaks. In contrast, engagement with ADHD-related posts in Spanish was much lower than in English and only slightly exceeded that of ASD at its peak in 2022. ADHD likes per post differed significantly between English- and Spanish-language posts (*P*<.001 [Kruskal-Wallis test]). Anxiety, SUD, and insomnia showed significant engagement, peaking during the pandemic. Depression, schizophrenia, and suicide followed similar trends with an increase in likes around 2020 to 2021.

**Figure 3:**
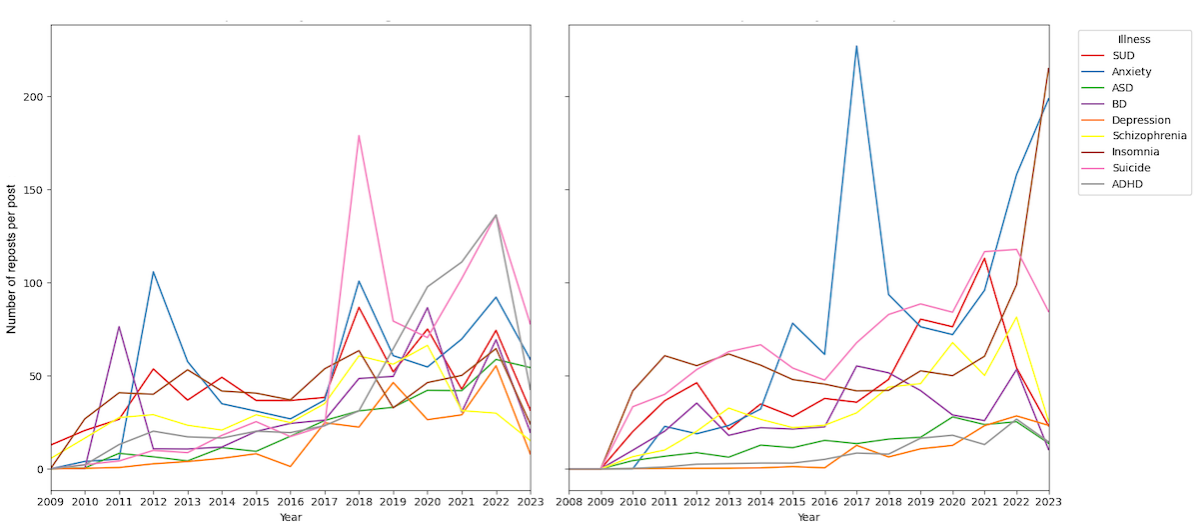
Number of reposts per post over time for various mental health conditions in both English (left) and Spanish (right). ADHD: attention-deficit/hyperactivity disorder; ASD: autism spectrum disorder; BD: bipolar disorder; SUD: substance use disorder.

Figure 3 presents line graphs illustrating the number of reposts per post over time for various mental health conditions. Overall, there was a noticeable increase in reposts per post over time. Reposts per post differed significantly across conditions in both English and Spanish posts (*P*<.001 [Kruskal-Wallis test]). In English-language posts, ADHD-related posts showed notable engagement, with a pronounced increase between 2018 and 2022. ASD-related posts did not receive the same level of attention, with engagement levels remaining relatively low. Suicide-related posts had the highest peak in engagement, with the number of reposts per post nearing 200 around 2018 and another significant but smaller peak around 2022. Anxiety-related posts also experienced a significant increase in engagement, with peaks around 2012, 2018, and 2022. SUD-related posts maintained steady engagement over the years, with noticeable peaks in 2018, 2020, and 2022.

In Spanish-language posts, ADHD-related posts showed increased engagement but did not reach the high levels observed in English. ASD-related posts remained less prominent in terms of engagement, similar to the pattern observed in English-language posts. In Spanish-language posts, anxiety-related posts showed the most significant increase in engagement, with reposts per post peaking in 2017 and again in 2022. A similar pattern was observed with insomnia-related posts. SUD-related posts also showed notable engagement, with a peak around 2021. Across the full study period, ADHD posts had higher reposts per post than ASD posts in both English and Spanish (both *P*<.001 [Kruskal-Wallis test]), and ADHD reposts per post differed significantly between English- and Spanish-language posts (*P*<.001 [Kruskal-Wallis test]).

### Types of Personal Experiences in Posts About ADHD and ASD Compared to Other Mental Illnesses

**Figure 4:**
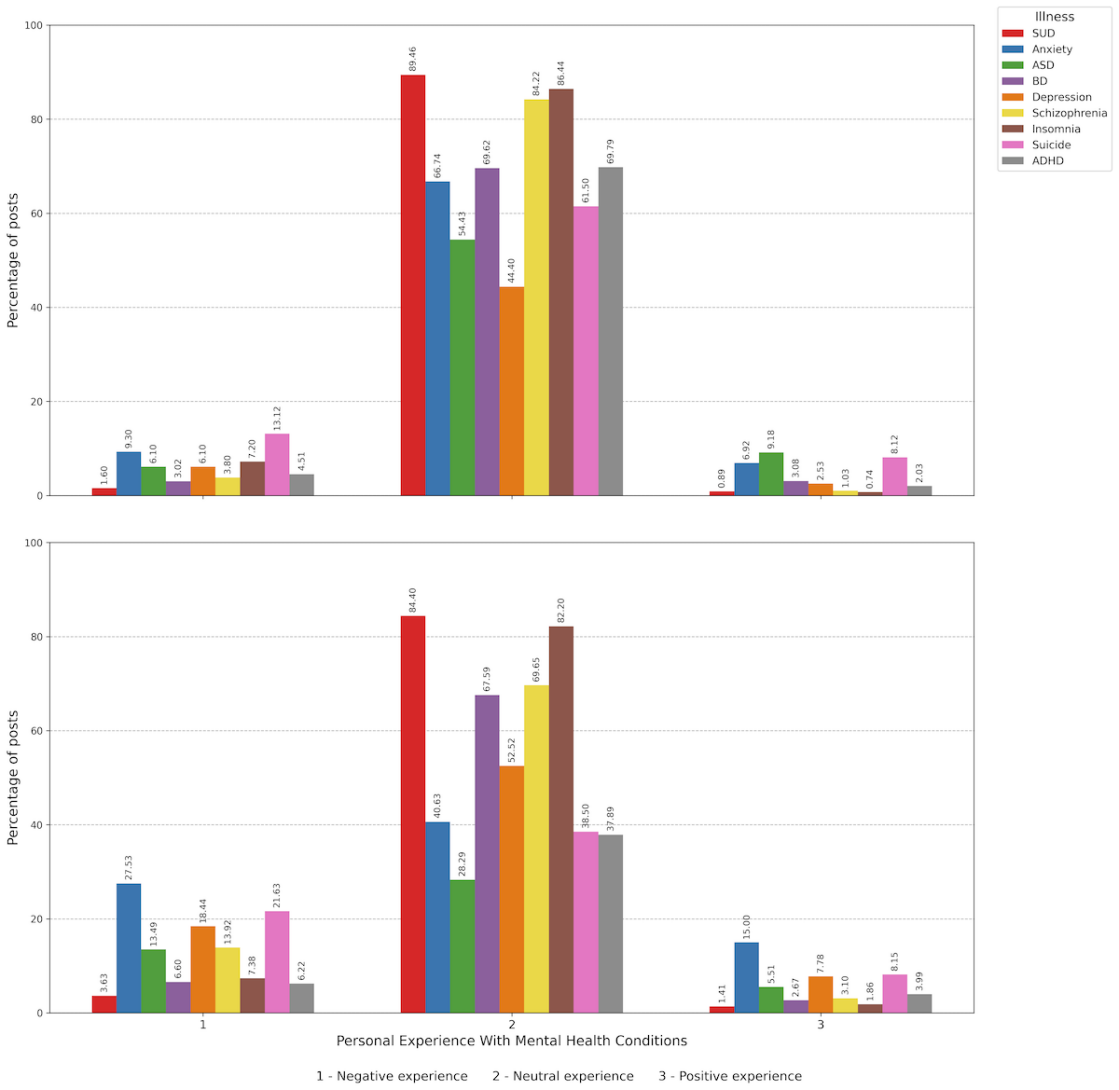
Distribution of posts that published personal experiences with mental health conditions. Top panel: posts in English. Bottom panel: posts in Spanish. ADHD: attention-deficit/hyperactivity disorder; ASD: autism spectrum disorder; BD: bipolar disorder; SUD: substance use disorder.

Figure 4 illustrates the distribution of posts sharing personal experiences. Among ADHD-related posts in English, 4378 of 97,084 (4.51%) posts described negative experiences, including stigmatization; 67,755 (69.79%) were neutral; and 1971 (2.03%) conveyed positive experiences. For ASD, 4552 of 74,619 (6.10%) posts indicated negative experiences; 40,615 (54.43%) were neutral; and 6850 (9.18%) were positive.

Among other mental illnesses, including anxiety, bipolar disorder, depression, insomnia, SUD, schizophrenia, and suicide, the distribution followed a similar trend, with an average of 6.30% of negative experiences (SD 3.99%, range 1.60%-13.13%); 71.77% of neutral experiences (SD 16.17%, range 44.38%-89.46%); and 3.33% of positive experiences (SD 3.01%, range 0.74%-8.12%).

When comparing languages, Spanish-language posts about ADHD showed a higher percentage of both negative (1133/18,223, 6.22% vs 4378/97,084, 4.51%) and positive (727/18,223, 3.99% vs 1971/97,084, 2.03%) experiences but fewer neutral experiences (37.89% vs 69.79%) than English posts. Similarly, Spanish-language posts about ASD contained more negative (6674/49,475, 13.49% vs 4552/74,619, 6.1%) experiences but fewer positive (2726/49,475, 5.51% vs 6850/74,619, 9.18%) and neutral (13,996/49,475, 28.29% vs 40,615/74,619, 54.43%) experiences than their English counterparts. Spanish posts were more polarized, showing more negative and positive experiences, while English posts were more often neutral. Language differences in the distribution of personal experience categories were statistically significant for both ADHD and ASD (both chi-square test *P*<.001). This pattern was consistent across various mental illnesses.

### Trivialization of ADHD and ASD in Posts Compared to Other Mental Illnesses

**Figure 5:**
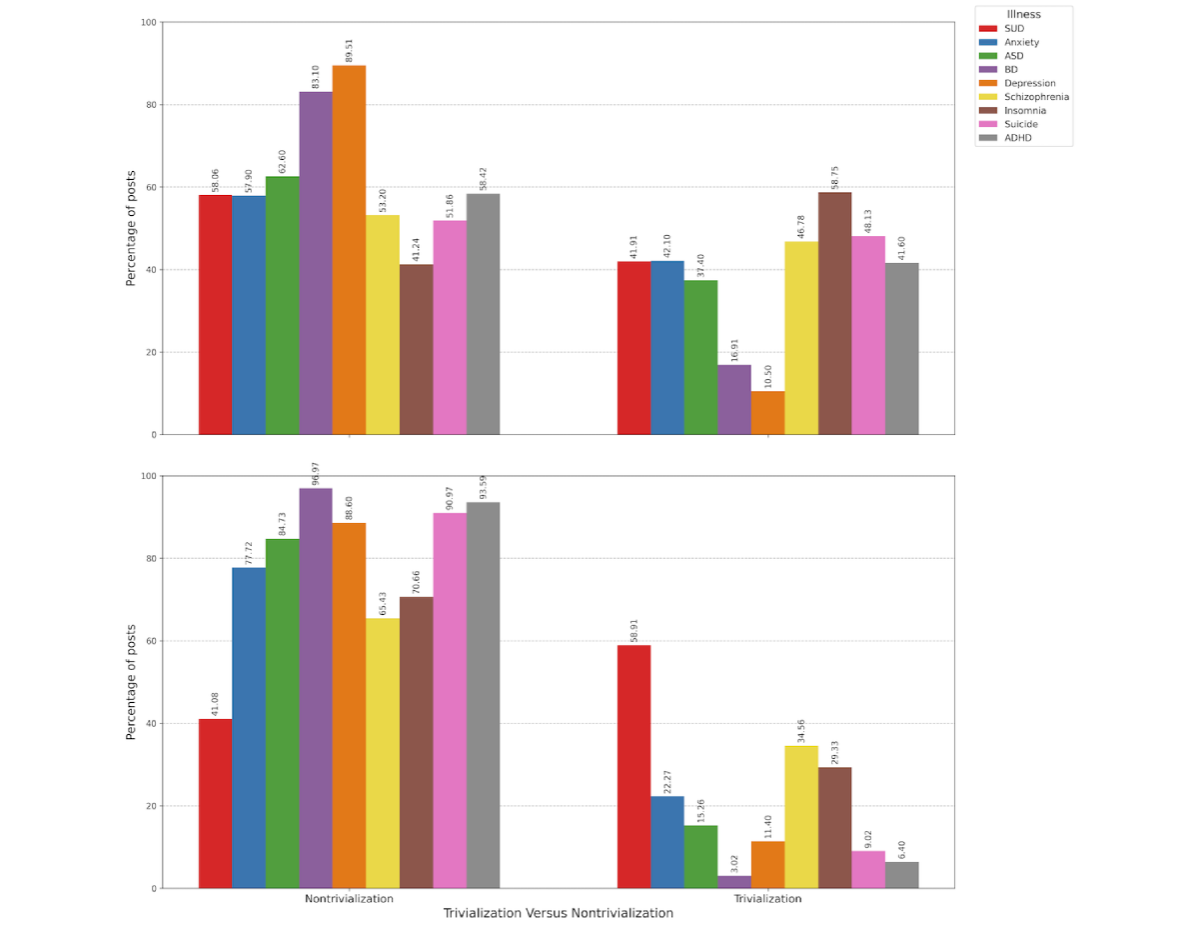
Comparison of nontrivializing vs trivializing posts about mental health conditions. Top panel: posts in English. Bottom panel: posts in Spanish. ADHD: attention-deficit/hyperactivity disorder; ASD: autism spectrum disorder; BD: bipolar disorder; SUD: substance use disorder.

Figure 5 shows that nontrivializing posts were more common overall, with this trend being even more pronounced in Spanish-language posts. In English-language posts, nontrivializing posts accounted for an average of 61.77% (SD 15.24%, range 41.24%-89.51%). ADHD- and ASD-related posts followed this pattern, with 56,716 of 97,084 (58.42%) posts and 46,711 of 74,619 (62.60%) posts being nontrivializing, respectively.

In Spanish-language posts, the percentage of nontrivializing posts was higher (mean 78.86%, SD 17.67%, range 41.08%-96.97%). For ADHD- and ASD-related posts, nontrivializing posts accounted for 17,055 of 18,223 (93.59%) posts and 41,933 of 49,475 (84.73%) posts, respectively, with significant differences between English- and Spanish-language posts for both conditions (both chi-square test *P*<.001).

### User-Perceived Causes of Mental Illness Analysis

**Figure 6:**
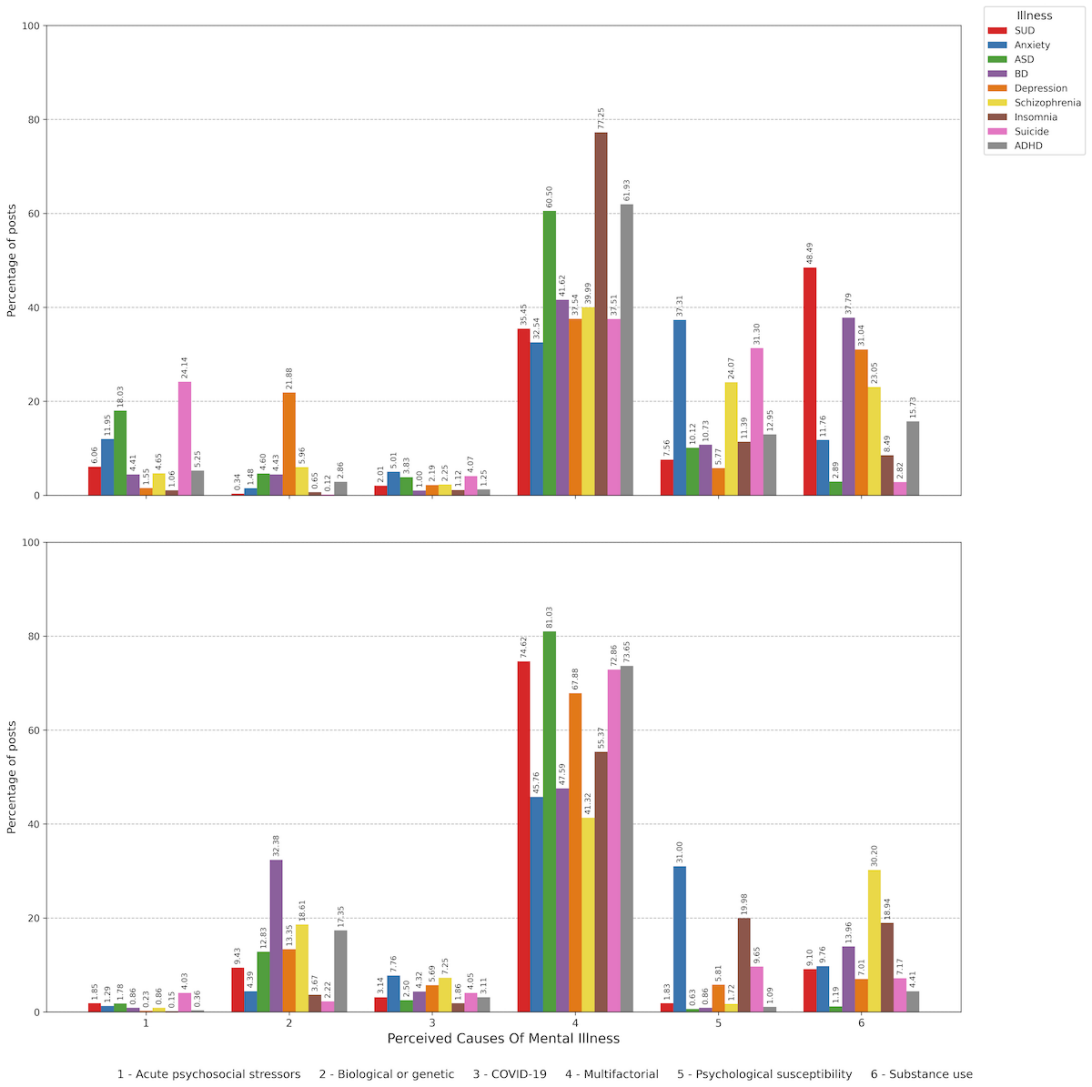
User-perceived causes of mental illness. Top panel: posts in English. Bottom panel: posts in Spanish. ADHD: attention-deficit/hyperactivity disorder; ASD: autism spectrum disorder; BD: bipolar disorder; SUD: substance use disorder.

Figure 6 illustrates user-perceived causes of mental illness. For ADHD in English-language posts, the most commonly attributed causes were multifactorial factors (60,124/97,084, 61.93%), followed by substance use (15,281/97,084, 15.74%) and psychological susceptibility (12,572/97,084, 12.95%). For ASD-related posts, the leading causes were multifactorial factors (45,144/74,619, 60.5%); acute psychosocial stressors (13,461/74,619, 18.04%); and psychological susceptibility (7551/74,619, 10.12%). Notably, substance use was associated with ADHD in 15,281 of 97,084 (15.74%) English-language posts, whereas in Spanish-language posts, this association was much lower (805/18,223, 4.42%). For other psychiatric conditions in English-language posts, multifactorial causes were the most frequently cited for anxiety (32,775/100,723, 32.54%); bipolar disorder (7517/18,061, 41.62%); depression (2055/5473, 37.55%); insomnia (10,880/14,082, 77.26%); SUD (8930/25,190, 35.45%); schizophrenia (31,791/79,478, 40%); and suicide (36,326/96,817, 37.52%). Psychological susceptibility was the most common perceived cause for anxiety (37,590/100,723, 37.32%) and was the second most common cause for schizophrenia (19,130/79,478, 24.07%) and suicide (30,313/96,817, 31.31%). Unsurprisingly, substance use was the most frequently cited cause for SUD (12,215/25,190, 48.49%), while it was also commonly mentioned in bipolar disorder–related posts (6827/18,061, 37.8%). Overall, the distribution of perceived-cause categories differed significantly between ADHD and ASD in English-language posts (chi-square test *P*<.001).

In Spanish-language posts, there were some differences in perceived causes. For ADHD, the most attributed causes were multifactorial (13,421/18,223, 73.65%), followed by biological or genetic factors (3162/18,223, 17.35%) and substance use (805/18,223, 4.42%). For ASD, the leading causes were multifactorial (40,095/49,475, 81.04%), biological or genetic factors (6353/49,475, 12.84%), and COVID-19 (1237/49,475, 2.5%). For other psychiatric conditions in Spanish-language posts, multifactorial causes were the most frequently cited, with higher percentages than in English-language posts for SUD (5606/7512, 74.63%); suicide (33,670/46,205, 72.87%); depression (9517/14,019, 67.89%); insomnia (39,077/70,561, 55.38%); bipolar disorder (1763/3703, 47.6%); anxiety (48,416/105,780, 45.77%); and schizophrenia (10,751/26,019, 41.32%). Biological or genetic factors were the second most frequently cited cause for bipolar disorder (1199/3703, 32.39%) and SUD (709/7512, 9.44%), while substance use was the second most cited cause for schizophrenia (7860/26,019, 30.21%). Psychological susceptibility was most frequently cited for anxiety (32,802/105,780, 31.01%) but was attributed less frequently to other conditions. Overall, the distribution of perceived-cause categories differed significantly between ADHD and ASD in Spanish-language posts (chi-square test *P*<.001).

When comparing languages, the distribution of perceived-cause categories differed significantly between English- and Spanish-language posts for both ADHD and ASD (both chi-square test *P*<.001).

### Percentage of User-Perceived Treatability of Mental Illnesses in Posts About ADHD and ASD vs Other Mental Illnesses

**Figure 7:**
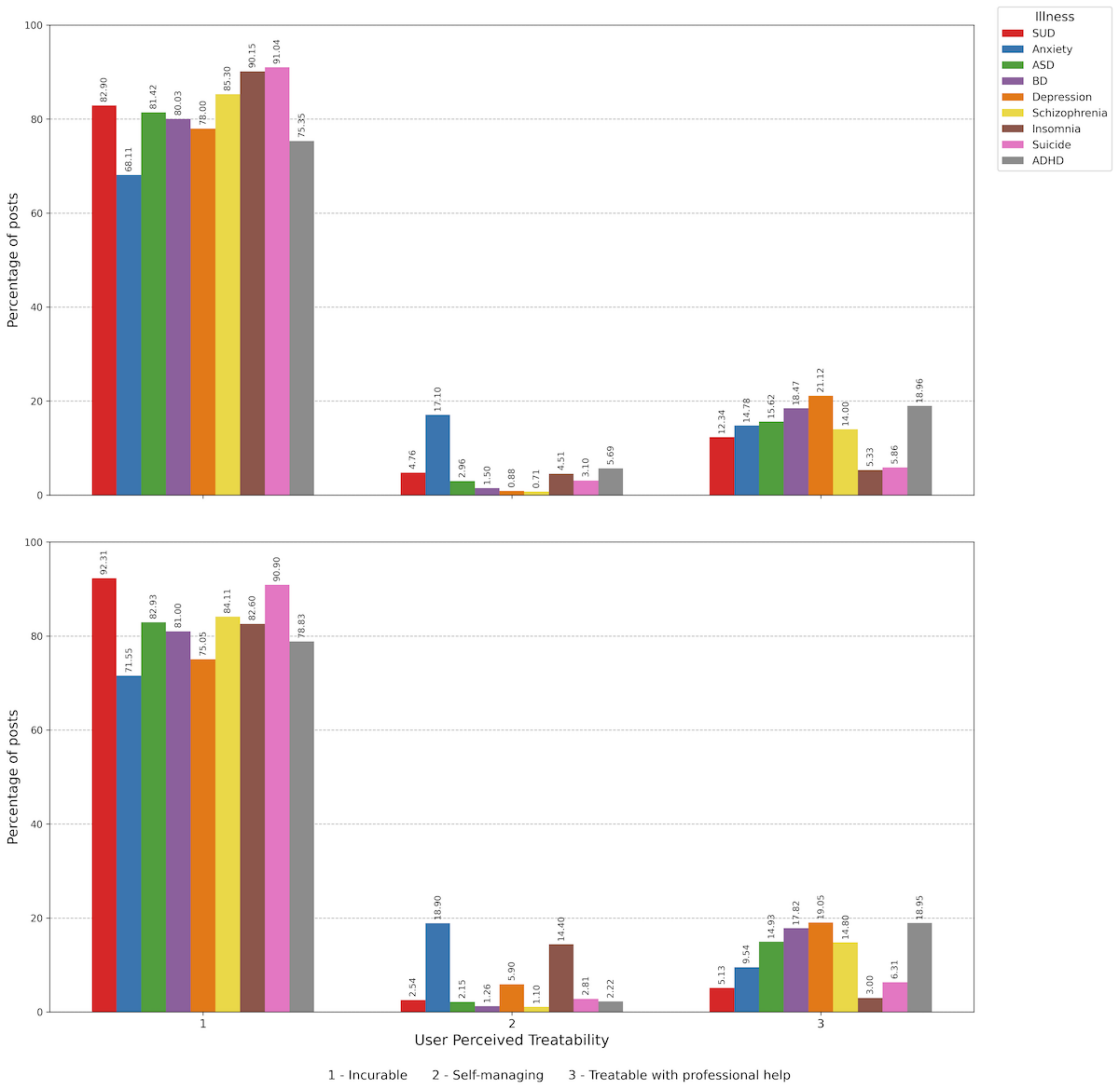
User-perceived treatability of mental illnesses. Top panel: posts in English. Bottom panel: posts in Spanish. ADHD: attention-deficit/hyperactivity disorder; ASD: autism spectrum disorder; BD: bipolar disorder; SUD: substance use disorder.

Figure 7 illustrates the user-perceived treatability of mental illnesses. Among English-language posts about ADHD, 73,153 of 97,084 (75.35%) users view it as incurable; 18,407 (18.96%) describe it as treatable with professional help; and 5524 (5.69%) mention that it can be self-managed. For ASD, the perception of incurability was even higher at 60,755 of 74,619 (81.42%) posts, with 11,655 (15.62%) mentioning it is treatable with professional help and 2209 (2.96%) referring to it as self-manageable. The distribution of treatability categories differed between ADHD and ASD posts in English (chi-square test *P*<.001). For other psychiatric conditions, English-language posts reflect a high perception of incurability (mean 82.22%, SD 7.88%, range 68.12%-91.04%). The perception that these conditions are treatable with professional help averages 13.13% (SD 5.92%, range 5.33%-21.12%), while self-manageability averages 4.65% (SD 5.73%, range 0.71%-17.10%). Anxiety stands out, with 17,224 of 100,723 (17.10%) users stating it can be self-managed, significantly higher than the 3.01% average for other conditions when anxiety was excluded (chi-square test *P*<.001).

In Spanish-language posts, some differences emerged. For ADHD, 14,365 of 18,223 (78.83%) users perceived it as incurable, slightly higher than the sentiment in English-language posts, while 3453 of 18,223 (18.95%) users believed it is treatable with professional help. The perception of self-manageability was lower (405/18,223, 2.22%). For ASD, the perception of incurability was even higher (41,030/49,475, 82.93%), while 7387 (14.93%) believed it was treatable with professional help, and only 1064 (2.15%) thought it can be self-managed. The distribution of treatability categories differed between ADHD and ASD posts in the Spanish language (chi-square test *P*<.001). For other psychiatric conditions, Spanish-language posts indicated a similarly high perception of incurability at 82.49% (SD 7.60%, range 71.56%-92.32%). However, the perception of treatability with professional help was lower at 10.81% (SD 6.43%, range 2.99%-19.05%), while the perception of self-manageability was slightly higher at 6.70% (SD 7.10%, range 1.10%-18.90%). When comparing languages, the distribution of treatability categories differed significantly between English- and Spanish-language posts for both ADHD and ASD (both chi-square test *P*<.001).

## Discussion

### Principal Findings

The study used machine learning techniques to analyze data from X on public perceptions of ADHD and ASD, with other mental illnesses included as comparators. Our analysis of 852,990 posts revealed a substantial increase in post volume and engagement across the observation period. Notably, ADHD-related posts in English experienced a marked surge in engagement (likes and reposts) during the COVID-19 pandemic, with likes per post exceeding 1200 between 2020 and 2023. In contrast, ASD consistently received lower engagement in both English- and Spanish-language posts. Personal experiences differed by language, with ADHD posts being predominantly negative in both languages and ASD posts being mostly positive in English but largely negative in Spanish. Overall, posts conveyed a nontrivializing attitude toward neurodevelopmental disorders, particularly in Spanish. Perceived causes also varied, with English-language posts frequently citing substance use and psychological factors, while Spanish-language posts emphasized biological or genetic causes, with substance use cited as a secondary factor for ADHD and COVID-19 cited as a secondary factor for ASD. Users generally viewed mental health conditions as chronic with limited optimism for treatment; fewer than 7% believed in self-management, except for anxiety. Professional help was rarely endorsed, although ADHD was viewed as more treatable than ASD. These insights can inform clinicians and researchers about public attitudes and experiences regarding neurodevelopmental disorders, particularly ADHD and ASD, underscoring the value of social media analysis in providing real-time, unfiltered, and empirically derived data in areas where traditional research methods may encounter limitations.

The findings suggest growing public interest in ADHD during the analyzed period, while ASD received comparatively less attention [25]. Diagnostic criteria changes in *Diagnostic and Statistical Manual of Mental Disorders, 5th Edition* (2013) [26], including older age of onset, fewer symptom requirements for adults, and addition of age-appropriate clinical examples, likely contributed to the increase in ADHD awareness and prevalence [27,28]. Social media proliferation of ADHD content may have further boosted public engagement [29]. Furthermore, the COVID-19 pandemic may have exacerbated ADHD symptoms, increasing recognition and diagnosis [30,31]. Butt et al [2] found that ADHD-related visits in Ontario increased by 32% compared with visit rates before the pandemic, especially among female individuals aged 5 to 9 and 20 to 55 years. Conversely, ASD awareness has grown but not to the extent of ADHD awareness, possibly due to individuals with autism favoring limited social interactions during the pandemic [32]. Pandemic lockdowns may have reduced social demands, easing certain stressors for individuals with ASD [33]. Thorell et al [34] reported that distance learning negatively impacted children with ADHD more than those with ASD. In the same study, families affected by ASD even reported some positive effects, such as reduced social pressure [34]. Additionally, it is possible that individuals with ASD participate more in private online communities that are not captured by mainstream social media [35,36].

While awareness of ADHD and ASD has increased, experiences shared online reveal persistent challenges. ADHD was associated predominantly with negative experiences in both languages, consistent with previous findings [7,37,38]. Mueller et al [7] noted significant ADHD-related stigma, including bullying and social withdrawal. ASD elicited mostly positive experiences in English-language posts but predominantly negative experiences in Spanish-language posts. Morgan et al [39] highlighted systemic barriers, including language, cultural obstacles, and issues with translated screening tools, limiting timely ASD diagnosis for Spanish speakers and increasing negative experiences. Griffiths et al [38] reported that adults with autism experienced higher rates of negative life events, such as abuse and financial exploitation, leading to increased anxiety, depression, and reduced life satisfaction. Similarly, Cooper et al [37] found that individuals with autism had lower self-esteem and higher depression and anxiety compared with peers without autism. Families also reported stigma and discrimination, negatively impacting their mental health and ability to provide support [40].

It is unclear whether increased awareness of ADHD and ASD has significantly reduced stigma and trivialization [3,4,7]. Social media has likely enhanced visibility, empathy, and acceptance through authentic portrayals and advocacy [29,41]. Educational initiatives and the more recent neurodiversity movement also reinforced positive attitudes [42]. Dillenburger et al [12] found ASD awareness among school peers increased from 50% in those aged 11 years to 80% in those aged 16 years; half knew someone with autism, while about 3% self-identified as autistic. However, Alsehemi et al [43] found that although 88% of respondents had heard of ASD, 41% admitted limited understanding.

Although most users endorsed multifactorial causes, the perceived contributing factors for ADHD and ASD varied by language. English-language posts often linked mental illness to substance use and psychological vulnerability, while Spanish-language posts emphasized biological and genetic explanations. ADHD was frequently described as multifactorial in English-language posts, with substance use and psychological factors cited, aligning with the literature on ADHD’s complex genetic and environmental etiology [44]. In contrast, Spanish-language posts primarily attributed both ADHD and ASD to biological or genetic causes, with secondary mentions of substance use for ADHD and COVID-19 for ASD. These findings align with research on ADHD’s complex etiology, involving genetic and environmental influences [44]. Other studies have also noted frequent user emphasis on behavioral outcomes, such as self-medication and mental health struggles [45]. The perception of ASD as multifactorial echoes findings by Mitchell and Locke [46], who reported that the public often cites genetic and neurological causes for this condition.

Our study found the public typically views mental health conditions as chronic, with limited optimism about treatment outcomes. However, ADHD is uniquely seen as more treatable, comparable to depression, likely reflecting its documented responsiveness and generally improved prognosis into adulthood [47]. Conversely, ASD is perceived as less amenable to significant improvement. The limited interest in professional support expressed in posts contrasts with other findings, such as Angermeyer et al [48], who reported high public regard for professional help. This discrepancy may result from survey biases, including social desirability—respondents providing socially acceptable answers—and selection bias, as more engaged individuals typically participate. Anxiety was an exception regarding perceived self-manageability. This may be due to its high lifetime prevalence of 28.8% [49] and broad public awareness [50]. The perception may also be influenced by the widespread availability of self-help resources, including books, websites, and apps [51], as well as by the demonstrated effectiveness of nonpharmacological interventions such as cognitive behavioral therapy, which can often be self-administered [52,53].

Using X offers advantages over traditional surveys, which may suffer from response distortion and nonresponse bias [8,9]. X provides real-time, unsolicited opinions from a broad user base, potentially yielding more naturalistic and representative insights into public views on mental health. This study has limitations. X users may not represent the general population, as they skew younger and more tech-savvy [54]. Only English- and Spanish-language posts were analyzed, excluding other language groups. Language is an imperfect proxy for geography and health systems; we interpreted differences as discourse differences rather than as country-level differences. Keyword-based collection may have missed relevant posts, and despite data cleaning, misclassification and noise remain risks. Our keyword strategy did not include legacy-diagnostic terms (eg, Asperger); posts using only these terms may be undercaptured, particularly in earlier years of the study period. Public sentiment here refers to publicly available discourse on the platform. We did not reliably separate individual users from professional or institutional accounts, which may influence observed discourse patterns. Analyses were conducted at the post level rather than the individual user level; therefore, comention of multiple conditions reflects discourse overlap and cannot be interpreted as clinical comorbidity. Individual experiences based solely on text may miss emotional nuance, and the study’s observational design limits causal interpretations. Future research should expand to additional social media platforms and incorporate a broader range of languages to capture more diverse perspectives. Incorporating advanced sentiment analysis techniques, such as context-aware and emotion-specific models, could further deepen our understanding of public attitudes toward mental health.

### Conclusions

This study applied machine learning to data obtained from X to examine public perceptions of ADHD and ASD. ADHD-related content saw a surge in engagement during the COVID-19 pandemic, while ASD-related content received less attention. Although most posts were neutral, negative experiences—particularly for ADHD—were more common. Users generally viewed mental health conditions as chronic and difficult to treat, with fewer than 7% expressing belief in self-management, except for anxiety. In English-language posts, perceived causes of ADHD and ASD were largely multifactorial, often citing substance use and psychological factors. Spanish-language posts followed similar patterns in tone but emphasized biological and genetic factor explanations.

These insights can inform clinicians and researchers about public attitudes and experiences regarding neurodevelopmental disorders, particularly ADHD and ASD, underscoring the value of social media analysis in capturing real-time, unfiltered, and empirically derived data—especially in areas where traditional research methods may be limited by response biases or restricted reach.

## Abbreviations

ADHD: attention-deficit/hyperactivity disorder
ASD: autism spectrum disorder
BERTweet: Transformer-Based Language Model Pretrained on English Posts
BETO: Transformer-Based Language Model Pretrained on Spanish Text
EDA: Easy Data Augmentation
SUD: substance use disorder
